# Combination Effect of Outdoor Activity and Screen Exposure on Risk of Preschool Myopia: Findings From Longhua Child Cohort Study

**DOI:** 10.3389/fpubh.2021.607911

**Published:** 2021-03-05

**Authors:** Lihua Huang, Katrina L. Schmid, Xiao-Na Yin, Jingyu Zhang, Jianbo Wu, Guiyou Yang, Zeng-Liang Ruan, Xiao-Qin Jiang, Chuan-An Wu, Wei-Qing Chen

**Affiliations:** ^1^Department of Medical Statistics and Epidemiology, Guangzhou Key Laboratory of Environmental Pollution and Health Assessment, Guangdong Provincial Key Laboratory of Food, Nutrition and Health, School of Public Health, Sun Yat-sen University, Guangzhou, China; ^2^School of Optometry and Vision Science, Faculty of Health, Queensland University of Technology, Brisbane, QLD, Australia; ^3^Longhua District Maternal and Child Health Hospital, Shenzhen, China; ^4^Department of Information Management, Xinhua College of Sun Yat-sen University, Guangzhou, China

**Keywords:** screen use, outdoor activity, myopia, early childhood, interaction

## Abstract

Evidence regarding screen use and outdoor activity during very early childhood (i. e., from aged 1 to 3 years) and their potential combined links to the later preschool myopia is limited. This information is needed to release effective public health messages and propose intervention strategies against preschool myopia. We collected information regarding very early childhood screen use, outdoor activity and the kindergartens vision screenings of 26,611 preschoolers from Longhua Child Cohort Study by questionnaires. Logistic regression models were used to examine the associations between reported outdoor activity, screen use from 1 to 3 years of age, and preschool myopia. Throughout very early childhood, from 1 to 3 years, the proportion of children exposed to screens increased (from 35.8 to 68.4%, *p* < 0.001), whereas the proportion of children who went outdoors ≥7 times/week (67.4–62.1%, *p* < 0.001) and who went outdoors for ≥60 min/time (53.3–38.0%, *p* < 0.001) declined. Exposure to fixed screen devices [adjusted odds ratio (AOR) = 2.66, 95% confidence interval (CI) = 2.09–3.44], mobile screen devices (AOR = 2.76, 95% CI = 2.15–3.58), and limited outdoor activity (AOR = 1.87, 95% CI = 1.42–2.51) during early childhood were associated with preschool myopia. Among children whose parents were myopic, the interactions between outdoor activity and fixed or mobile screen use on later preschool myopia were significant; the ORs and 95% CI were 3.34 (1.19–9.98) and 3.04 (1.06–9.21), respectively. Our findings suggest the possibility that the impact of screen exposure during early childhood on preschool myopia could be diminished by outdoor activity for children whose parents have myopia.

## Introduction

Myopia is a critical public health problem around the world. In 2050, myopia prevalence is projected to reach 49.8% (4,758 million) globally, and early-onset myopia will be more common (1, 2). Having myopia caused by an elongated eye is associated with an increased risk of pathology causing visual impairment and blindness (3). This adds to the burden of disease associated with myopia (4) and reduced quality of life (5). Causes of myopia include both genetic and environmental factors (6). The earlier myopia starts, the faster myopia progresses, and the greater the risk of high myopia later in life (7). Studies have shown that early childhood is an important period for visual development (8, 9). Therefore, the determination of modifiable risk factors for myopia that is present during early childhood is important for developing a feasible strategy to curb the growing prevalence of myopia. This is particularly the case for mainland China where the number of myopes is predicted to reach 700 million to 800 million, and the number of high myopes will reach 100 million to 200 million by the year 2100 without the implementation of effective myopia control measures (10).

Studies involving school-aged children suggest that near work can promote the incidence and progression of myopia (11). Regarding exposure to screen devices including televisions (TVs), computers, and smartphones, the evidence is variable across devices perhaps because of different working distances and screen sizes. For example, some studies report that the use of computers/video games and watching TV were significant risk factors for the progression of myopia among children (12), and more recently, electronic screen (i.e., smartphones) use was also suggested to be associated with myopia (13). However, several studies have not observed a significant association between TV viewing and myopia in school-aged children and adults (14–16). Also, a recent meta-analysis study of five studies concluded that digital screen time was not proven to be associated with myopia (17). Thus, the evidence of screen exposure as a risk factor for myopia among very young children remains limited.

Studies involving school-aged children show that outdoor activity appears to prevent or retard myopia (18). However, only a few studies in Singapore have explored the association between limited outdoor activity during early childhood and myopia and found no connections (19, 20). Their relationship has not been examined among the Chinese preschool population. Studies have suggested that outdoor activity might dilute the impact of near work on myopia or mean refraction (21, 22); however, whether it could moderate the association between screen use and myopia has been underexplored. Only one recent study has shown that the combined effect of near work (including computer use) could be decreased by outdoor exposure (23).

As both the growth of the eye and visual system development are rapid during early childhood (8, 9), it is necessary to verify the relationship of both early childhood screen use and outdoor activity during this critical period with later myopia, including the exploration of possible interaction of outdoor activity and screen use. The routine vision screening of kindergarten children in Longhua District of Shenzhen and the survey of Longhua Children Cohort Study (LCCS) in 2017 enabled the investigation of relationships between early childhood screen use (including fixed screen devices and mobile screen devices) and outdoor activity with myopia among preschool children.

## Materials and Methods

### Study Participants

Children and their families were recruited from the LCCS in 2017. LCCS is a prospective population-based study, which examined the influence of environmental factors surrounding children's early life on child psychobehavioral development; it commenced in September 2014 with once-yearly follow-up (24, 25). LCCS recruited the children when they entered kindergarten and asked their primary caregivers to complete a self-administered structured questionnaire every year. In the 2017 survey, a total of 29,595 caregivers of preschool children provided written informed content and completed a self-administered structured questionnaire regarding parental age at childbirth, parental education level, the refractive conditions of the parents (emmetropia, myopia, or other visual disorders), monthly household income, child's gender, and child's date of birth. Detailed questions on screen exposure and outdoor activity were included. Caregivers also provided the results of children's vision screening. Data of children who had missing information on either the sociodemographic characteristics or refractive condition or had been clinically diagnosed with another type of refractive error (i.e., non-emmetropia and non-myopia) were excluded. The survey data of 26,611 (89.9%) preschool children were analyzed. Supplementary Figure 1 presents the flow diagram of the participant selection process. The study was approved by the Ethics Committee of the School of Public Health at Sun Yat-sen University (ethics clearance no. 2015-016), and the legal guardians of all participants provided informed consent. All methods were performed under relevant guidelines and regulations.

### Determination of Screen Exposure

The questionnaire included a set of questions to collect information regarding screen exposure from 1 to 3 years of age.

Q1: Was your child involved in watching TV or computers or other fixed electronic screens during the year of age 1? Two options were provided: “0” = “no,” “1” = “yes.”
*Q2:* Was your child involved in using smartphones, tablets, and other mobile electronic screens during the year of age 1? Two options were provided: “0” = “no,” “1” = “yes.”

The questions were repeated for each age band (i.e., 1–2 years and 2–3 years). This information was converted into three variables to describe fixed screen use, mobile screen use, and total screen use (yes/no) when children were aged 1 to 3 years. As long as it was reported that the child had any screen exposure during this time, his/her status of screen use was regarded as “yes;” otherwise, screen exposure status was deemed a “no.”

### Determination of Outdoor Activity

The questionnaire included a set of questions concerning the outdoor activity of the children from 1 to 3 years of age.

*Q1:* How often was your baby taken outside during the year of age 1? Two options were available: “0” = “≥7 times/week” and “1” = “ <7 times/week”
*Q2:* How long per time was your baby outside on average?” Two options were available: “0” = “ <60 min” and “1” = “≥60 min.”

The questions were repeated for each age band (i.e., 1–2 years and 2–3 years). The answers were used to generate the following variables that described the children's outdoor activity during the year of age 1 to 3: (1) the frequency of outdoor activity, (2) the duration of outdoor activity, and (3) the overall outdoor factors, which included different possible combinations of the frequency and duration of the outdoor activity (e.g., high frequency and long duration, low frequency and short duration).

### Determination of Myopia

As shown in the previous studies (26–28), the vision screening for preschool children is performed by ophthalmologists from Longhua District Maternal and Child Health Hospital twice per school year, since 2017. The results of the vision screening are conveyed to children's caregivers by teachers. If the results of screening indicate abnormal refraction, parents are advised to take the child for a more comprehensive eye examination at the ophthalmic clinic of Longhua District Maternal and Child Health Hospital (27). The ophthalmologists from Longhua District Maternal and Child Health Hospital defined the children's refractive problem as myopia if the spherical equivalent was equal to or worse than −0.50 D in at least one eye (27). In the present study, therefore, primary caregivers completed the survey questions about whether the child had been diagnosed with any types of refractive error based on the written report of the vision screening visit (26–28). The final analysis included only myopic and emmetropic children.

### Covariates

Potential covariates include child's age, gender, parental age at childbirth, level of parental education (junior high school and below, high school or technical secondary school, junior college, and graduate and above), monthly household income (<5,000, 5,000–10,000, 10,000–15,000, 15,000–20,000, and ≥20,000 RMB per month) and parental history of myopia (no, at least one parent had myopia). These factors have been shown to either influence the amount of screen time and outdoor activity children perform or myopia risk (29–31).

### Statistical Analysis

Descriptive statistics (means and standard deviations) have been used to describe continuous variables and absolute frequencies and proportions to describe categorical variables. Student *t* test and χ^2^ test were applied to assess the difference in demographic characteristics between (1) children whose data were included vs. excluded in this study and (2) myopic children and emmetropic children whose data were included in the final analysis.

Logistic regression models with and without the adjustment of covariates were utilized to evaluate the associations between total screen use, fixed and mobile screen use, frequency and duration of outdoor activity, overall outdoor factor, and myopia, respectively. And unadjusted odds ratio (OR) and adjusted OR (AOR) and their 95% confidence intervals (95% CIs) were reported, respectively. The correlation and the colinearity of the potential covariates were evaluated using the cross-correlation matrix (Spearman coefficient) and variance inflation factor, respectively. Covariates included in the adjustment model were as follows: child's age, gender, maternal age at childbirth, monthly household income, and paternal history of myopia. In addition to including these covariates, outdoor activity and screen exposure have been mutually adjusted in the respective models.

To examine the interaction of screen exposure and outdoor activity on myopia, multiplicative interaction analyses with adjustment for the potential confounders were performed. To facilitate interpretation of their interaction, subgroup analyses were conducted, with data of children who had no screen exposure and who performed high levels of outdoor activity (i.e., high frequency and/or long duration) used as the reference group.

To determine the relationships of screen exposure and outdoor activity with myopia among children whose parents having myopia and children whose parents not having myopia, the data of children were stratified based on the reported status of parental myopia, and the analyses above were repeated.

The analyses were performed using R statistical software (version 3.4.0, http://www.r-project.org), with two-sided *p* < 0.05 required for significance.

## Results

### Summary of Demographic Characteristics

Compared with children whose data were excluded from this study (*n* = 2,984), 26,611 (89.9%), included data were from children who were younger (4.6 vs. 4.7 years, *p* < 0.001), and the proportion with a family history of myopia was lower (40.5 vs. 59.3%, *p* < 0.001). Other characteristics were comparable between the two groups, including parental education, parental age, family income, children's age, and children's myopia status (Supplementary Table 1).

Table 1 depicts the comparison of demographic characteristics between myopic children and emmetropic children. Of 26,611 children included in this analysis, 604 children (2.3%) were myopic. Myopic children were slightly older (4.9 vs. 4.6 years, *p* < 0.001), and the proportion with myopic parents was higher (63.7 vs. 40.0%, *p* < 0.001), compared with emmetropic children. Except for the uneven proportion of family income among the refractive groups, other characteristics were comparable, including gender, parental age at childbirth, and parental education (Table 1).

**Table 1:** Comparison of demographic characteristics between emmetropic children and myopic children.

**Characteristic**	**Emmetropic children (*n* = 26,007)**	**Myopic children (*n* = 604)**	***P*-value**
**Age (years)**	4.6 (0.9)	4.9 (0.8)	<0.001
**Gender**			
Male	14,088 (54.2)	352 (58.3)	0.050
Female	11,919 (45.8)	252 (41.7)	
**Maternal education**			
Junior high school and below	6,491 (25.0)	170 (28.1)	0.153
High school or technical secondary school	7,650 (29.4)	181 (30.0)	
Junior college	6,362 (24.5)	144 (23.8)	
Graduate and above	5,504 (21.2)	109 (18.0)	
**Paternal education**			
Junior high school and below	5,369 (20.6)	151 (25.0)	0.073
High school or technical secondary school	7,040 (27.1)	156 (25.8)	
Junior college	5,947 (22.9)	133 (22.0)	
Graduate and above	7,651 (29.4)	164 (27.2)	
**Family income (Yuan/month)**			
<5,000	3,823 (14.7)	104 (17.2)	0.019
5,000–10,000	6,813 (26.2)	174 (28.8)	
10,000–15,000	4,974 (19.1)	111 (18.4)	
15,000–20,000	3,621 (13.9)	91 (15.1)	
≥20,000	6,776 (26.1)	124 (20.5)	
Maternal age at childbirth	27.1 (4.2)	27.4 (4.6)	0.178
**Paternal age at childbirth**	29.7 (4.8)	29.8 (5.1)	0.731
**Parental history of myopia**			<0.001
No	15,612 (60.0)	219 (36.3)	
Yes (at least one parent with myopia)	10,395 (40.0)	385 (63.7)	

*Mean (SD) are presented for continuous variables, and N (%) are presented for non-continuous variables*.

### Amount of Screen Use and Outdoor Activity From 1 to 3 Years of Age

**Figure 1:**
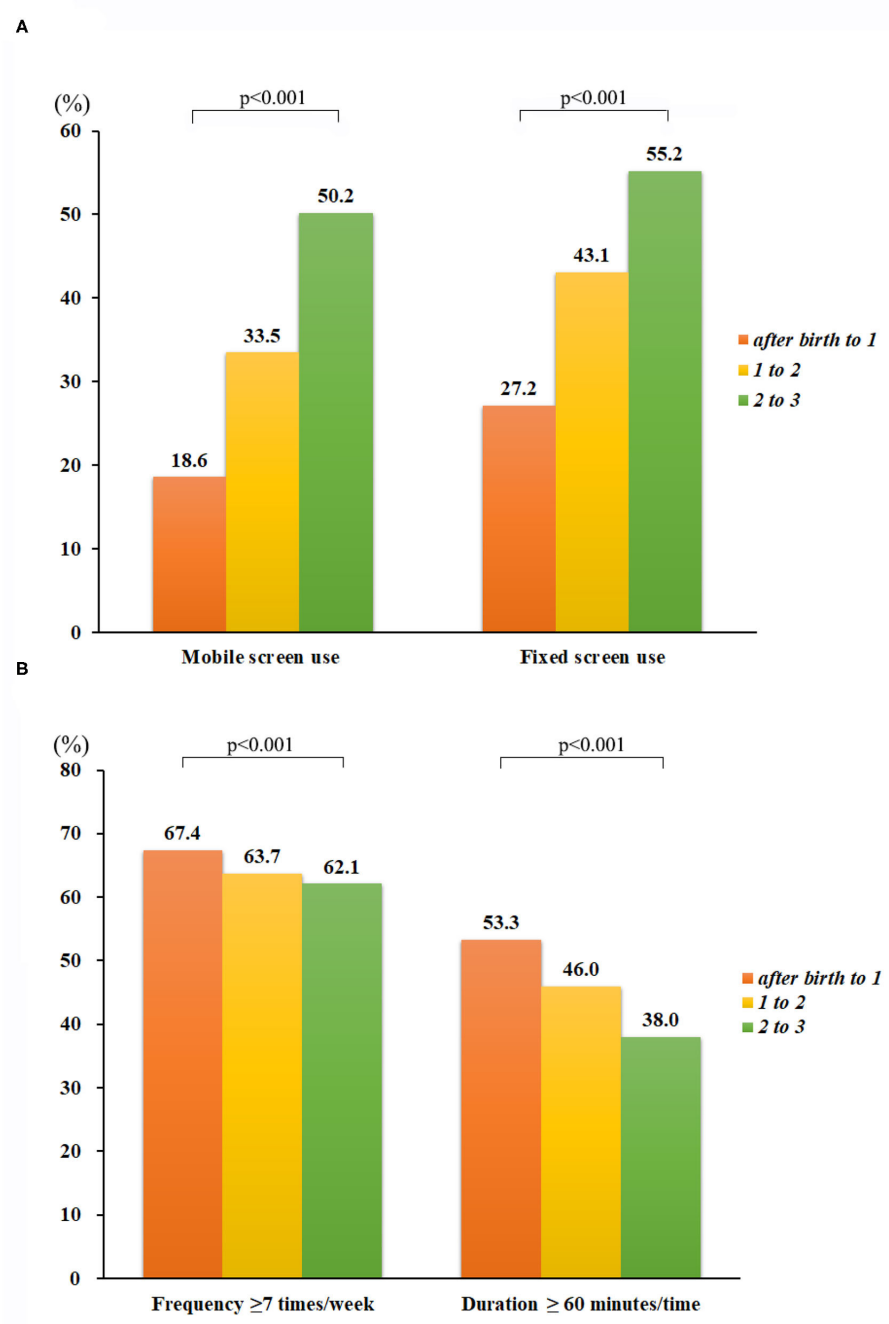
Yearly outdoor activity and screen use from 1 to 3 years of age. During the first 3 years after birth, the proportion of children who went outdoors for ≥7 times/week declined, from 67.4% at age 1 year to 62.1% at age 3 years, as did the proportion of children who spent ≥60 min outdoors, from 53.3% at age 1 year to 37.4% at age 3 years. In contrast, the proportion of children exposed to fixed screen devices increased from 17.7% at age 1 year to 55.3% at age 3 years, and the proportion of children exposed to handheld mobile devices went from 18.6% at age 1 year to 40.2% at age 3 years.

During the 1st year of life, 9,531 (35.8%, in Supplementary Table 2) children had been provided screen-based devices to use. Specifically, 7,364 (27.7%) children had access to fixed screen devices (e.g., TV and computers), and 4,948 (18.6%) children had been provided small handheld mobile devices (e.g., smartphones and tablets). More than half of children, 17,939 (67.4%), went outside very frequently (≥7 times/week), with 14,177 (53.3%) children reporting spending large amounts of time (≥60 min) outdoors (Figure 1). During the 2nd year of life, slightly more than half of the children (*n* = 14,209, 53.4%) had been exposed to screens, whereas 63.7% (*n* = 16,946) of children reported frequent periods spent outdoors, and 46.0% (*n* = 12,242) were outside for long durations (Figure 1 and Supplementary Table 2). By 2 to 3 years of age, the proportion of children who had used screen-based devices had increased to 68.4% (*n* = 18,212). Specifically, 55.2% (*n* = 14,692) of children had used fixed screen devices, and 50.2% (*n* = 13,353) of children had used mobile screen devices, whereas the proportion of children going outdoors frequently declined (to 62.1%, *n* = 16,523, *p* < 0.001), as had the proportion of children who spent long periods outdoors (38.0%, *n* = 10,107, *p* < 0.001) (Figure 1 and Supplementary Table 2).

### Relationships of Screen Use and Outdoor Activity From 1 to 3 Years of Age With Preschool Myopia

The child's age, gender, maternal age at childbirth, monthly household income, and parental history of myopia were selected as the covariates (in Supplementary Tables 3, 4). In addition, outdoor activity and screen exposure have been mutually adjusted in the respective models. After adjusting for these covariates, compared with no exposure to screens before 3 years of age, exposures to all screen devices, fixed screen devices, and mobile screen devices were significantly associated with preschool myopia (AOR for total screen use: 2.50, 95% CI = 1.96–3.22, in Supplementary Table 5; AOR for fixed screen use: 2.66, 95% CI = 2.09–3.44; AOR for mobile screen use: 2.76, 95% CI = 2.15–3.58; Table 2). Compared with children reported to frequently go outdoors, those who had less frequent outdoor activity were linked with preschool myopia (AOR = 1.75, 95% CI = 1.43–2.16; Table 2), and compared with children who spent long durations of time outdoors, those who spent shorter durations outdoors were associated with preschool children (AOR = 1.25, 95% CI = 1.05–1.48; Table 2). In addition, compared with children who were reported to have both a high frequency and a long duration of outdoor activity, children who went outside relatively infrequently were related to preschool myopia (AORs = 1.51–1.87; Table 2). The stratified analyses based on parental myopia status showed similar associations (Table 2).

**Table 2:** Association between screen use and outdoor activity from 1 to 3 years of age and preschool myopia.

**Characteristic**	**All**		**Children with non-myopic parents (*n* = 15,831)**	**Children with myopic parents (*n* = 10,780)**
	**OR (95% CI)**	**AOR^a^ (95% CI)**	**AOR^b^ (95% CI)**	**AOR^b^ (95% CI)**
**Screen use**				
Fixed screen use (e.g., TV)				
No screen use	Ref.	Ref.	Ref.	Ref.
Yes	2.61 (2.06, 3.36)^***^	2.66 (2.09, 3.44)^***^	2.98 (2.06, 4.45)^***^	2.45 (1.79, 3.44)^***^
Mobile screen use (e.g., smartphones and tablets)				
No screen use	Ref.	Ref.	Ref.	Ref.
Yes	2.52 (1.98, 3.25)^***^	2.76 (2.15, 3.58)^***^	3.17 (2.17, 4.78)^***^	2.49 (1.81, 3.51)^***^
**Outdoor activity**				
Frequency				
≥7 times/week	Ref.	Ref.	Ref.	Ref.
<7 times/week	1.69 (1.38, 2.08)^***^	1.75 (1.43, 2.16)^***^	1.70 (1.18, 2.51)^**^	1.77 (1.38, 2.29)^***^
Duration				
≥6 min/time	Ref.	Ref.	Ref.	Ref.
<60 min/time	1.28 (1.08, 1.52)^**^	1.25 (1.05, 1.48)^*^	1.53 (1.14, 2.07)^**^	1.12 (0.91, 1.39)
Overall outdoor factors				
≥7 times/week + ≥6 min/time	Ref.	Ref.	Ref.	Ref.
≥7 times/week + <6 min/time	1.08 (0.74, 1.56)	0.99 (0.68, 1.44)	1.18 (0.59, 2.36)	0.93 (0.60, 1.45)
<7 times/week + ≥6 min/time	1.51 (1.12, 2.07)^**^	1.51 (1.12 2.08)^**^	1.38 (0.80, 2.52)	1.59 (1.11, 2.33)^*^
<7 times/week + <6 min/time	1.88 (1.43, 2.52)^***^	1.87 (1.42, 2.51)^***^	2.09 (1.28, 3.66)^**^	1.77 (1.27, 2.53)^**^

*OR, odds ratio; AOR, adjusted odds ratio; CI, confidence interval*.

* *p < 0.05*,

** *p < 0.01*,

*** *p < 0.001*.

a *Regarding associations between screen exposure and myopia, the models were adjusted for children's age, gender, maternal age at childbirth, monthly household income, parental history of myopia (myopic, non-myopic), and outdoor activity, whereas regarding associations between outdoor activity and myopia, the models were adjusted for children's age, gender, maternal age at childbirth, monthly household income, parental history of myopia (myopic, non-myopic), and screen exposure*.

b *Regarding associations between screen exposure and myopia, the models were adjusted for children's age, gender, maternal age at childbirth, and monthly household income, and outdoor activity, whereas regarding associations between outdoor activity and myopia, the models were adjusted for children's age, gender, maternal age at childbirth, monthly household income, and screen exposure*.

### Interaction of Screen Use and Outdoor Activity From 1 to 3 Years of Age on Preschool Myopia

**Figure 2:**
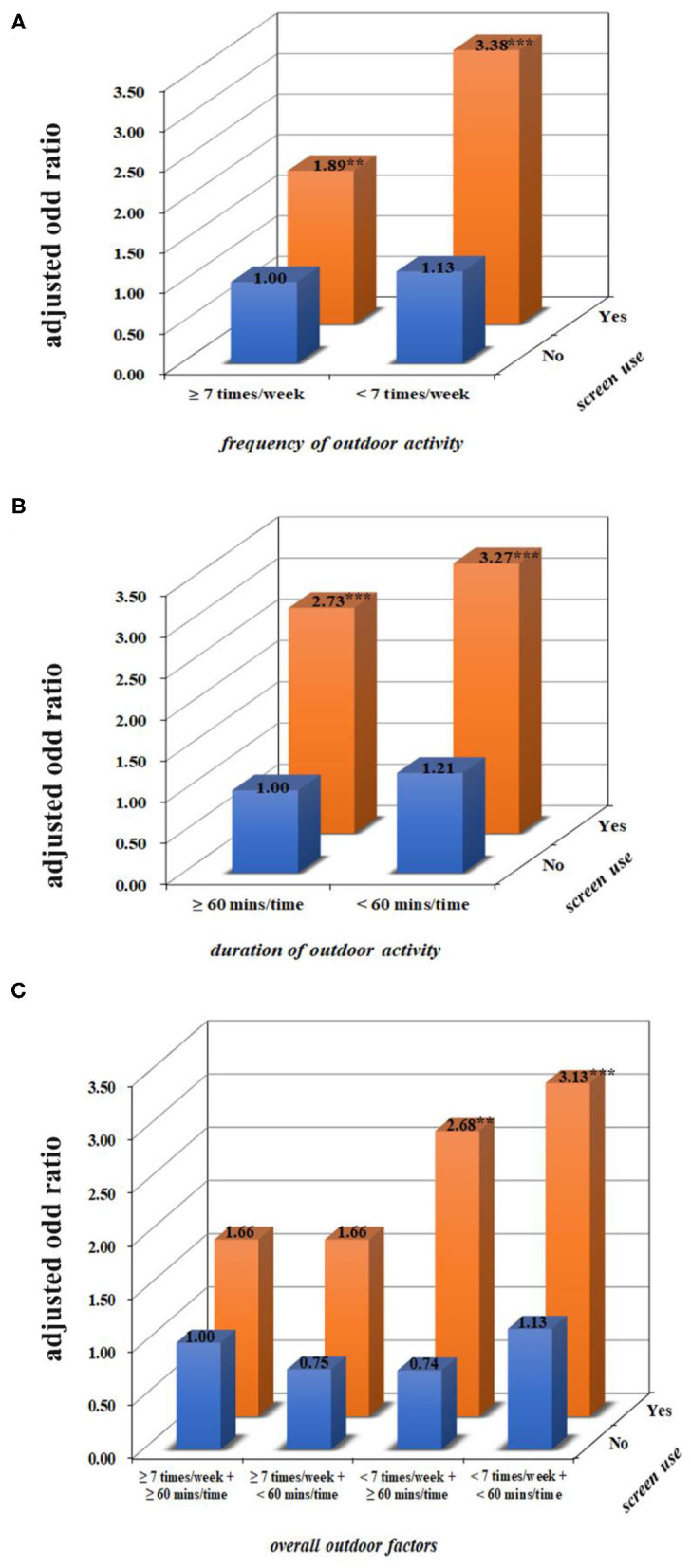
The combined effect of fixed screen use and outdoor activity from 1 to 3 years of age on preschool myopia. **(A–C)** Present the combined effects of frequency, duration of outdoor activity, the overall outdoor factors, and screen use on myopia after adjusting for children's age, gender, maternal age at childbirth, monthly household income, and parental history of myopia. Compared with children who had not been exposed to screens and had a high level of outdoor activity, children who were reported to have exposure to fixed screen devices and a low frequency of outdoor activity had an increased prevalence of myopia. **p < 0.01, ***p < 0.001.

**Figure 3:**
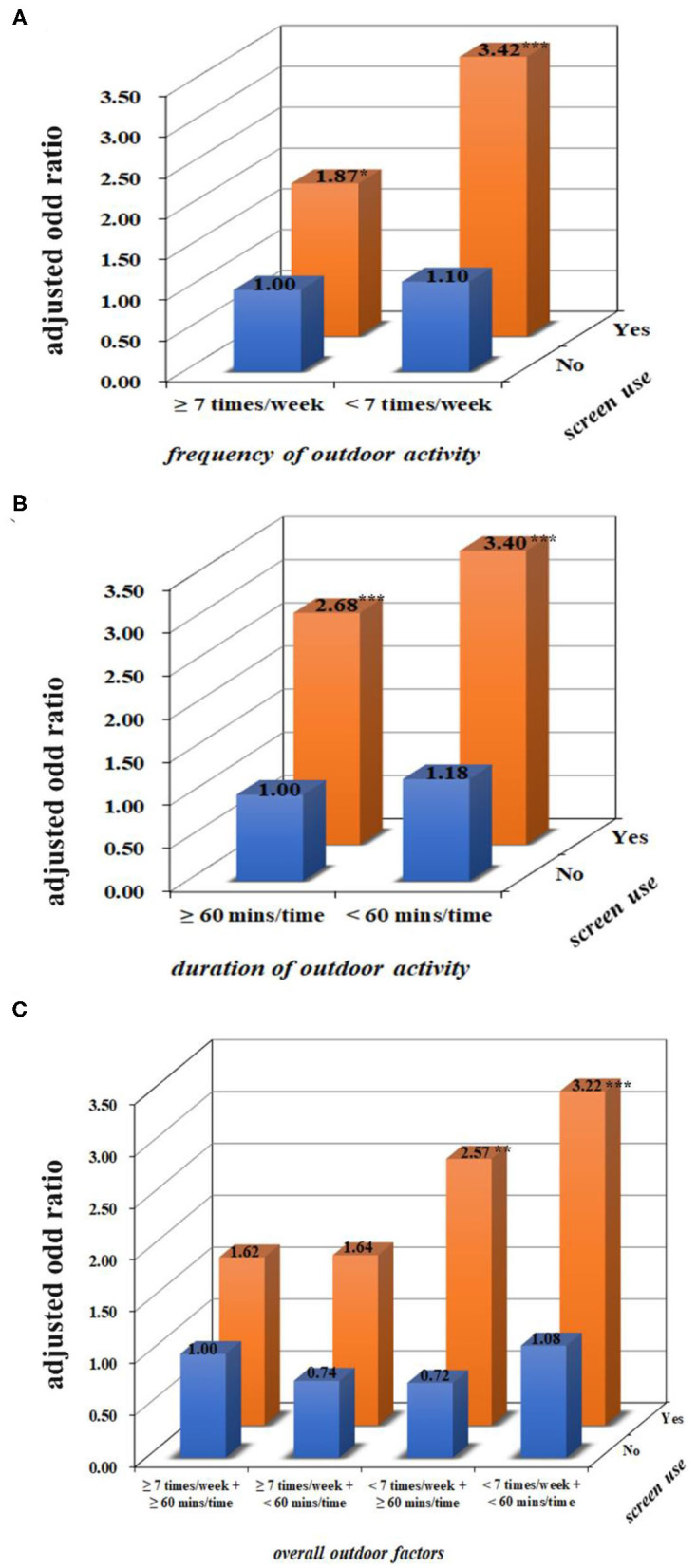
The combined effect of mobile screen use and outdoor activity from 1 to 3 years of age on preschool myopia. **(A–C)** Present the combined effects of frequency, duration of outdoor activity, the overall outdoor factors, and screen use on myopia after adjusting for children's age, gender, maternal age at childbirth, monthly household income, and parental history of myopia. Compared with children who had not been exposed to screens and had a high level of outdoor activity, children who were reported to have exposure to mobile screen devices and a low frequency of outdoor activity had an increased prevalence of myopia. ^*^p < 0.05, ^**^p < 0.01, ^***^p < 0.001.

After adjusting for the child's age, gender, maternal age at childbirth, monthly household income, and parental history of myopia, no interactions between outdoor activity and fixed screen use were significant (*p* > 0.05; Table 3 and Figure 2). However, after the data were stratified based on parental myopia status, one significant interaction was observed for children who had a family history of myopia (Table 3). To be specific, compared with children who had not been exposed to fixed screens and had reported both a high frequency and a long duration of outdoor activity, myopia prevalence was higher in children who had been both exposed to fixed screens and also reported less frequent, but still long duration of outdoor activity (AOR = 2.21, 95% CI = 1.22–4.44; Table 3). There was also a relatively high prevalence of myopia in children who had been exposed to fixed screens and reported less frequent and shorter durations of outdoor activity (AOR = 2.30, 95% CI = 1.29–4.54; Table 3), although compared to children who had not been exposed to fixed screens and reported a high frequency with long duration of outdoor activity, the risk difference was not statistically significant. Similar results were observed in the interaction of mobile screen use and outdoor activity on preschool myopia (Table 4 and Figure 3), and in the interaction of total screen use and outdoor activity on preschool myopia (Supplementary Table 6).

**Table 3:** Interaction between fixed screen use and outdoor activity from 1 to 3 years of age on preschool myopia.

**Pattern**		**All**		**Children with non-myopic parents (*n* = 15,831)**	**Children with myopic parents (*n* = 10,780)**
		**OR (95% CI)**	**AOR^A^ (95% CI)**	**AOR^B^ (95% CI)**	**AOR^B^ (95% CI)**
**Fixed screen use**	**Frequency of outdoor activity**				
No screen use	≥7 times/week	Ref.	Ref.	Ref.	Ref.
No screen use	<7 times/week	1.04 (0.64, 1.75)	1.13 (0.69, 1.91)	1.37 (0.60, 3.69)	1.06 (0.57, 2.02)
Yes	≥7 times/week	1.80 (1.14, 2.95)^*^	1.89 (1.20, 3.11)^**^	2.57 (1.12, 6.92)^*^	1.65 (0.97, 2.99)
Yes	<7 times/week	3.01 (2.00, 4.77)^***^	3.38 (2.24, 5.37)^***^	4.16 (1.99, 10.66)^**^	3.08 (1.90, 5.36)^***^
Interaction	Screen use (yes) * frequency (<7times/week)	1.61 (0.91, 2.77)	1.58 (0.89, 2.72)	1.18 (0.41, 3.02)	1.76 (0.87, 3.48)
**Fixed screen use**	**Duration of outdoor activity**				
No screen use	≥60 min/time	Ref.	Ref.	Ref.	Ref.
No screen use	<60 min/time	1.23 (0.77, 2.01)	1.21 (0.75, 1.98)	1.65 (0.77, 3.93)	1.01 (0.55, 1.89)
Yes	≥60 min/time	2.60 (1.75, 4.03)^***^	2.73 (1.83, 4.24)^***^	3.35 (1.68, 7.65)^**^	2.41 (1.49, 4.12)^**^
Yes	<60 min/time	3.22 (2.20, 4.92)^***^	3.27 (2.23, 5.02)^***^	4.74 (2.46, 10.59)^***^	2.65 (1.67, 4.47) ^***^
Interaction	Screen use (yes)*duration (<6 min/time)	1.01 (0.59, 1.67)	0.99 (0.58, 1.65)	0.86 (0.34, 1.98)	1.09 (0.56, 2.09)
**Fixed screen use**	**Overall outdoor factors**				
No	≥7 times/week + ≥60 min/time	Ref.	Ref.	Ref.	Ref.
No	≥7 times/week + <60 min/time	0.81 (0.33, 1.89)	0.75 (0.31, 1.75)	2.36 (0.46, 17.06)	0.47 (0.15, 1.30)
No	<7 times/week + ≥60 min/time	0.70 (0.32, 1.53)	0.74 (0.34, 1.61)	1.74 (0.40, 11.90)	0.55 (0.20, 1.40)
No	<7 times/week + <60 min/time	1.07 (0.59, 2.09)	1.13 (0.62, 2.21)	2.44 (0.71, 15.34)	0.91 (0.44, 2.00)
Yes	≥7 times/week + ≥60 min/time	1.58 (0.87, 3.08)	1.66 (0.91, 3.24)	4.30 (0.99, 27.49)	1.20 (0.60, 2.53)
Yes	≥7 times/week + <60 min/time	1.70 (0.94, 3.29)	1.66 (0.91, 3.23)	4.02 (0.99, 25.93)	1.24 (0.64, 2.60)
Yes	<7 times/week + ≥60 min/time	2.44 (1.43, 4.55)^**^	2.68 (1.56, 5.02)^**^	5.19 (1.58, 32.02)^*^	2.21 (1.22, 4.44)^*^
Yes	<7 times/week + <60 min/time	2.92 (1.74, 5.38)^***^	3.13 (1.85, 5.78)^***^	7.60 (2.39, 46.24)^**^	2.30 (1.29, 4.54)^**^
Interaction	Screen use (yes) * outdoor activity (<6 min/time + ≥7times/week)	1.32 (0.51, 3.53)	1.35 (0.52, 3.61)	0.41 (0.05, 2.50)	2.24 (0.71, 7.92)
	Screen use (yes) * outdoor activity (≥6 min/time + <7times/week)	2.19 (0.93, 5.17)	2.19 (0.93, 5.18)	0.70 (0.09, 3.52)	3.34 (1.19, 9.98)^*^
	Screen use (yes) * outdoor activity (<6 min/time + <7times/week)	1.72 (0.82, 3.43)	1.68 (0.80, 3.37)	0.74 (0.11, 2.93)	2.11 (0.88, 4.89)

*OR, odds ratio; AOR, adjusted odds ratio; CI, confidence interval*,

* *p < 0.05*,

** *p < 0.01*,

*** *p < 0.001*.

a *Adjusted for children's age, gender, maternal age at childbirth, monthly household income, and parental history of myopia*.

b *Adjusted for children's age, gender, maternal age at childbirth, and monthly household income*.

**Table 4:** Interaction between mobile screen use and outdoor activity from 1 to 3 years of age on preschool myopia.

**Pattern**		**All**		**Children with non-myopic parents (*n* = 15,831)**	**Children with myopic parents (*n* = 10,780)**
		**OR (95% CI)**	**AOR^a^ (95% CI)**	**AOR^b^ (95% CI)**	**AOR^b^ (95% CI)**
**Mobile screen use**	**Frequency of outdoor activity**				
No screen use	≥7 times/week	Ref.	Ref.	Ref.	Ref.
No screen use	<7 times/week	1.04 (0.64, 1.75)	1.10 (0.67, 1.85)	1.35 (0.59, 3.64)	1.02 (0.55, 1.95)
Yes	≥7 times/week	1.63 (1.03, 2.70)^*^	1.87 (1.17, 3.10)^*^	2.45 (0.99, 6.75)	1.66 (0.96, 3.03)
Yes	<7 times/week	2.97 (1.97, 4.72)^***^	3.42 (2.26, 5.45)^***^	4.44 (2.11, 11.41)^***^	3.03 (1.86, 5.29)^***^
Interaction	Screen use (yes) ^*^ frequency (<7 times/week)	1.75 (0.98, 3.04)	1.66 (0.93, 2.90)	1.34 (0.45, 3.53)	1.79 (0.87, 3.57)
**Mobile screen use**	**Duration of outdoor activity**	
No screen use	≥60 min/time	Ref.	Ref.	Ref.	Ref.
No screen use	<60 min/time	1.23 (0.77, 2.01)	1.18 (0.74, 1.94)	1.63 (0.76, 3.88)	0.99 (0.54, 1.85)
Yes	≥60 min/time	2.41 (1.61, 3.76)^***^	2.68 (1.78, 4.18)^***^	3.63 (1.80, 8.35)^**^	2.24 (1.37, 3.85)^**^
Yes	<60 min/time	3.19 (2.17, 4.89)^***^	3.40 (2.31, 5.24)^***^	4.92 (2.53, 11.08)^***^	2.76 (1.73, 4.68)^***^
Interaction	Screen use (yes) ^*^ duration (<6 min/time)	1.04 (0.72, 1.49)	1.03 (0.71, 1.48)	0.84 (0.33, 1.95)	1.25 (0.64, 2.43)
**Mobile screen use**	**Overall outdoor factors**				
No screen use	≥7 times/week + ≥60 min/time	Ref.	Ref.	Ref.	Ref.
No screen use	≥7 times/week + <60 min/time	0.81 (0.33, 1.89)	0.74 (0.30, 1.73)	2.33 (0.45, 16.89)	0.46 (0.14, 1.28)
No screen use	<7 times/week + ≥60 min/time	0.70 (0.32, 1.53)	0.72 (0.33, 1.58)	1.72 (0.39, 11.75)	0.53 (0.19, 1.37)
No screen use	<7 times/week + <60 min/time	1.07 (0.59, 2.09)	1.08 (0.59, 2.12)	2.38 (0.69, 14.98)	0.87 (0.42, 1.90)
Yes	≥7 times/week + ≥60 min/time	1.45 (0.78, 2.85)	1.62 (0.88, 3.20)	4.05 (1.08, 26.26)	1.19 (0.59, 2.55)
Yes	≥7 times/week + <60 min/time	1.54 (0.83, 3.02)	1.64 (0.88, 3.23)	3.86 (0.99, 25.43)	1.24 (0.62, 2.64)
Yes	<7 times/week + ≥60 min/time	2.29 (1.33, 4.28)^**^	2.57 (1.49, 4.84)^**^	5.85 (1.77, 36.21)^*^	1.96 (1.07, 3.96)^*^
Yes	<7 times/week + <60 min/time	2.96 (1.76, 5.47)^***^	3.22 (1.91, 5.98)^***^	7.92 (2.48, 48.31)^**^	2.37 (1.33, 4.70)^**^
Interaction	Screen use (yes) * outdoor activity (<6 min/time + ≥7 times/week)	1.31 (0.50, 3.56)	1.36 (0.52, 3.71)	0.42 (0.05, 2.68)	2.23 (0.69, 8.01)
	Screen use (yes) ^*^ outdoor activity (≥6 min/time + <7times/week)	2.25 (0.95, 5.38)	2.19 (0.92, 5.26)	0.85 (0.11, 4.39)	3.04 (1.06, 9.21)^*^
	Screen use (yes) ^*^ outdoor activity (<6 min/time + <7times/week)	1.91 (0.90, 3.86)	1.83 (0.86, 3.73)	0.83 (0.12, 3.45)	2.27 (0.94, 5.36)

*OR, odds ratio; AOR, adjusted odds ratio; CI, confidence interval*,

* *p < 0.05*,

** *p < 0.01*,

*** *p < 0.001*.

a *Adjusted for children's age, gender, maternal age at childbirth, monthly household income, and parental history of myopia*.

b *Adjusted for children's age, gender, maternal age at childbirth, and monthly household income*.

## Discussion

From 1 to 3 years of age, the proportion of children exposed to screens (including fixed and mobile screens) increased, whereas the frequency and duration of outdoor activity declined. Using screen-based digital devices and going outside infrequently and/or only going outside for relatively short durations in early childhood were associated with preschool myopia. Outdoor activity moderated the association between screen use and preschool myopia among children whose parents reported being myopic. Our findings indicated that outdoor activity might lower the influence of screen exposure during very early childhood on preschool myopia among children whose parents are myopic.

Reduced outdoor time and increased indoor screen-viewing are common worldwide (32, 33). The findings here show that these behaviors commence very early in life, even before 3 years of age. This confirms previous findings showing a high prevalence of exposure to mobile devices in young children (34), and it has been reported that half of the preschool children in the United States were not taken outside to play daily (35). It has been suggested that children's physical activity today comprises less unstructured and unsupervised outdoor activity and more structured and supervised activity that primarily occurs indoors (36). This might be due to parents' heightened safety concerns and/or the limited outdoor spaces in urban cities (37, 38). Studies show that some parents use screen devices as a parenting tool to keep their children occupied, to keep their children calm in public places, during meals, and/ or even let their children use these as a bedtime aid (39, 40). These events lead to children being exposed to screens from an early age. These findings are alarming, because in general insufficient outdoor activity and excessive screen time are related to many health problems (i.e., obesity, psychological diseases and sleep disorder, etc.) (41, 42).

Evidence regarding the associations between exposure to screen devices including TVs, computers, and smartphones and myopia among school-aged children and adults is inconsistent. For example, the North India Myopia Study has suggested watching TV was a significant risk factor for the progression of myopia among urban school children (12), whereas several studies have not observed a significant association between TV viewing and myopia in either children or adults (14–16). Recently, digital electronic screen (i.e., computers, smartphones) usage was suggested to be associated with myopia (13). However, a recent meta-analysis study of five studies did not show a significant association between digital screen time and myopia prevalence and incidence (17). In agreement with some of the previous findings in school-aged children, our data for younger preschool children suggested that exposure to both fixed and mobile screen devices during the early life years, 1 to 3 years, was associated with preschool myopia. Our findings suggest the possibility that very young children were more sensitive to screen exposure including TV viewing, most likely because very early childhood is an important period for visual development (8, 9). Further studies are needed to verify our findings.

The relationships between insufficient outdoor time with myopia among school-aged children have previously been investigated. For example, a Danish cohort study of teenagers aged 16–17 years found that being physically active 3 h or more per week was associated with a marked decrease in myopia risk (43). A recent Taiwan study of students aged 6–20 years suggested that outdoor activities were associated with a lower risk of myopia (44). As for preschool-aged children, only a few studies from Singapore have explored the association between limited outdoor activity during early childhood and myopia and found a non-significant association (19, 20). Similar to the previous findings of school-aged children, our data for younger preschool children suggested that going outdoors for <7 times/week and/or <60 min/time during 1 to 3 years of age was inversely associated with preschool myopia. That our result was different from those studies involving Singapore preschool children might be due to (1) the different population and sample size, (2) the different study design, and (3) the different grouping of outdoor activity. Future studies are required to verify our findings and determine the exact timing and duration of the outdoor activities for the maximal reduction in myopia risk.

Subsequently, the possibility that varying levels of outdoor activity and screen exposure could interact to influence myopia was examined. It was observed that the combined effects of screen use and outdoor activity on preschool myopia were significant only for children with myopic parents. This finding is similar to a report that the combined effect of near work (including computer use) could be decreased by outdoor exposure (23). However, here only one significant interaction between screen exposure (yes) and low a level of outdoor activity (<7 times/week + ≥60 min/time) was observed. There might be many factors involved in why some measures that might be expected to be associated with myopia failed to reach statistical significance. First, the sample sizes across different environmental combinations varied. Second, the measurement of outdoor activity was based on parental reports, which may be inaccurate or biased. Questionnaires have been commonly used in studies assessing outdoor time in children, and these limitations were previously discussed (45); there is no other option for obtaining information on events that occurred in the past. Parental-reported measures of outdoor activity of their preschool-aged children are correlated (*r* = 0.3) with objective measures (i.e., a 3-dimensional accelerometer) (46). Third, based on the meta-analysis of 25 studies, relationship between increased time outdoors and the reduced risk of myopia might not be linear (18). Further studies are needed to verify our findings and the trend that outdoor activity moderated the association between screen use and preschool myopia. The significant interaction between screen use and outdoor activity on myopia among children whose parents have myopia not only highlights that the shared lifestyle and genetic factors within families play a vital role in myopia development, but also indicates that children of myopic parents might be more susceptible to these adverse environmental factors. As basic visual capacity is established shortly after birth and improves rapidly during the first few years (8, 9), it may be that frequent outdoor visual activities might be conducive to normal emmetropization. These findings collectively convey the public health message that young children should go outdoors daily for the purpose of lowering myopia risk, especially for the children whose parents have myopia. If the finding can be substantiated, it would have great potential public health significance because early-onset myopia is becoming more common, and these risk factors are modifiable.

The potential mechanisms for the link between increased outdoor activity and decreased myopia risk have been extensively considered. The high light intensity, the spectral composition of lighting, and the wavelengths of light present outdoors might increase retinal dopamine release and/or circulating vitamin D levels, thereby protecting the eye from elongation (47). The underlying mechanism for the association between screen exposure and myopia has also been studied, but no clear single mechanism was identified. As screen use is a form of near work, the mechanisms related to near work might help to explain the apparent risk that screen use brings (11, 48). Another possibility might be that outdoor activity promotes more frequent shifts of attention and focus, which is advantageous to the accommodation system (18). However, evidence on how visual activities could influence visual development during infancy and early childhood and thus the refraction at later ages is very limited; this is presumably mediated via the emmetropization process.

This work has several limitations to consider. First, data were obtained from children attending all kindergartens of Longhua District in Shenzhen, which is a very urbanized and densely populated city, which might limit the generalizability of the findings to other populations, particularly children living in rural areas. Second, selection bias might exist, because the proportion of myopic parents of the children excluded was higher than that of the children included in this study. Third, information was provided by parents and collected by a self-administered questionnaire, which might lead to unintentional reporting bias and thus influence the exact relationships with myopia. However, confidence in the current study findings is based on the previous use of these questionnaires and consistency with the findings of past studies (25, 26, 28, 49, 50). Fourth, myopia cases were identified based on the outcomes of a large sample vision screening and if necessary further eye testing and diagnosis at an ophthalmic clinic and then reported by primary caregivers; the number of steps involved in this process has the potential to lead to misclassification of refraction problems. However, the prevalence of myopia in this study and other studies in China that measured and recorded refraction during research visits is similar [i.e., Shanghai (51), and Guangzhou (52)]. Moreover, our previous findings regarding risk factors of myopia and astigmatism were consistent with findings from other studies that recorded cycloplegic refraction (13, 26, 28, 53). Additionally, as the original refraction data were not available, we were unable to explore the influence of screen exposure and outdoor activity on the amount of myopia and also cannot provide a further analysis of risk based on per diopter of myopia calculation. Fifth, although the results were corrected for children's age, gender, maternal age at childbirth, monthly household income, and parental history of myopia, we acknowledged other variables such as viewing distance, other types of near work (e.g., time of parental writing, reading), and genetic factors (in addition to the family history of myopia), which were not available for this study sample, which may contribute to residual confounding. Sixth, given the cross-sectional design of this study, the data do not address causality, but associations instead.

Nevertheless, our study provided a valuable reference regarding early-life screen use and outdoor activity and their possible associations with preschool myopia, which might interest clinicians and researchers alike because it was at this age that children's behavior patterns were shaped for the future, in relation not only to myopia but also to many aspects of their life and health. Changing children's lifestyles in this digital era requires action from all of those involved in the care of children. A good starting strategy would be not to use screen devices as a parenting tool or toy. Limiting screen use and going outdoors frequently might protect preschool children from early myopia. As myopia progresses most rapidly in younger children, this could have a great impact on the ultimate amount of myopia that develops (54). It could also provide a buffer to reduce the impact of upcoming educational pressure and increased near work on myopia (55). Further birth cohort studies are needed to verify the effect of avoiding screen use and going outdoor more frequently on myopia risk and also to explore the underlying mechanism.

## Conclusion

Our findings suggest that both very early childhood exposure to fixed and mobile screen devices and a lower level of outdoor activity were associated with the later preschool myopia, and outdoor activity moderated the influence of screen use particularly for children whose parents were myopic. Our findings point out the possibility that a high level of outdoor activity might offset preschool myopia risk related to screen use during early childhood. Future studies are required to verify this hypothesis, which would have great public health implications for myopia prevention in the current electronic age.

## Data Availability Statement

The raw data supporting the conclusions of this article will be made available by the corresponding author on reasonable request, without undue reservation.

## Ethics Statement

The studies involving human participants were reviewed and approved by the Ethics Committee of the School of Public Health at Sun Yat-sen University (ethics clearance No. 2015–016) and the legal guardians of all participants provided informed consent. All methods were performed in accordance with relevant guidelines and regulations. Written informed consent to participate in this study was provided by the participants' legal guardian/next of kin.

## Author Contributions

LH, X-NY, C-AW, and W-QC: study concept and design. LH, X-NY, JZ, KS, JW, GY, Z-LR, X-QJ, C-AW, and W-QC: acquisition, analysis or interpretation of data, statistical analysis and drafting of the manuscript. LH, KS, C-AW, and W-QC: critical revision of the manuscript for important intellectual content. All the authors reviewed the manuscript.

## Conflict of Interest

The authors declare that the research was conducted in the absence of any commercial or financial relationships that could be construed as a potential conflict of interest.
