# Supplementary material for: Combination Effect of Outdoor Activity and Screen Exposure on Risk of Preschool Myopia: Findings From Longhua Child Cohort Study
Source: Front Public Health. 2021 Mar 5;9:607911. doi: 10.3389/fpubh.2021.607911 (PMC7973103; doi:10.3389/fpubh.2021.607911)
Supplement: Supplementary file 1 [file Data_Sheet_1.docx]

Supplementary Material


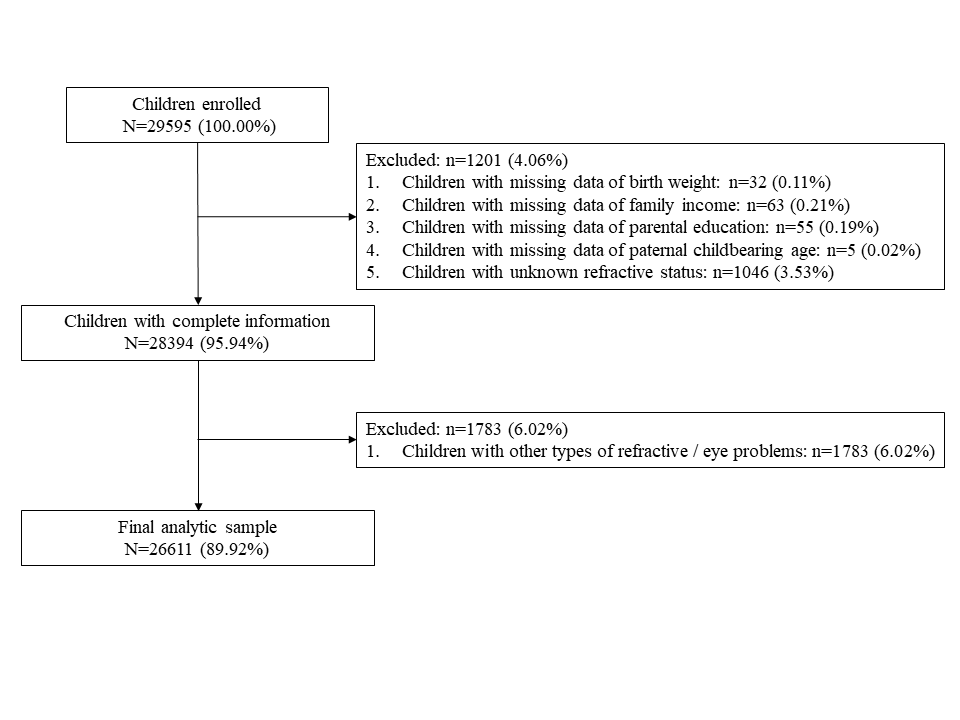


**Supplementary Figure 1 The flow diagram of the selection of participants**

**Supplementary Table 1.** **Comparison of demographic characteristics between children included and excluded in the final analysis**

| Characteristic | Children excluded (N=2984) | Children included (N=26611) | P-value |
| --- | --- | --- | --- |
| Age (years) | 4.7 (0.9) | 4.6 (0.9) | <0.001 |
| Gender |  |  |  |
| male | 1634 (54.8) | 14440 (54.3) | 0.620 |
| female | 1350 (45.2) | 12171 (45.7) |  |
| Maternal Education |  |  |  |
| Junior high school and below | 722 (24.9) | 6661 (25.0) | 0.054 |
| High school or technical secondary school | 787 (27.2) | 7831 (29.4) |  |
| Junior college | 737 (25.5) | 6506 (24.4) |  |
| Graduate and above | 648 (22.4) | 5613 (21.1) |  |
| Paternal Education |  |  |  |
| Junior high school and below | 565 (19.6) | 5520 (20.7) | 0.255 |
| High school or technical secondary school | 771 (26.7) | 7196 (27.0) |  |
| Junior college | 700 (24.3) | 6080 (22.8) |  |
| Graduate and above | 847 (29.4) | 7815 (29.4) |  |
| Family income (Yuan/month) |  |  |  |
| <5000 | 435 (14.9) | 3927 (14.8) | 0.213 |
| 5000~10000 | 809 (27.7) | 6987 (26.3) |  |
| 10000~15000 | 574 (19.7) | 5085 (19.1) |  |
| 15000~20000 | 377 (12.9) | 3712 (13.9) |  |
| ≥20000 | 725 (24.8) | 6900 (25.9) |  |
| Maternal age at childbirth | 27.0 (4.2) | 27.2 (4.2) | 0.482 |
| Paternal age at childbirth | 29.6 (4.9) | 29.7 (4.8) | 0.150 |
| Child’s myopia status |  |  |  |
| Emmetropia | 144 (98.0) | 26005 (97.7) | 0.999 |
| Myopia | 3 (2.0) | 604 (2.3) |  |
| Parental history of myopia |  |  |  |
| No | 1213 (40.7) | 15831 (59.5) | <0.001 |
| Yes (at least one parent with myopia) | 1771 (59.3) | 10780 (40.5) |  |

Mean (SD) are presented for continuous variables and N (%) are presented for non-continuous variables.

**Supplementary Table 2. Screen use and outdoor activity from 1 to 3 years of age**

| Characteristic | N (%) | | | p-value |
| --- | --- | --- | --- | --- |
|  | During the year of age 1 | During the year of age 2 | During the year of age 3 |  |
| Screen use |  |  |  |  |
| Total screen use |  |  |  | <0.001 |
| No | 17080 (64.2) | 12402 (46.6) | 8399 (31.6) |  |
| Yes | 9531 (35.8) | 14209 (53.4) | 18212 (68.4) |  |
| Fixed screen use |  |  |  | <0.001 |
| No | 19247 (72.3%) | 15136 (56.9) | 11919 (44.8) |  |
| Yes | 7364 (27.7%) | 11475 (43.1) | 14692 (55.2) |  |
| Mobile screen use |  |  |  | <0.001 |
| No | 21663 (81.4) | 17700 (66.5) | 13258 (49.8) |  |
| Yes | 4948 (18.6) | 8911 (33.5) | 13353 (50.2) |  |
| Outdoor activity |  |  |  |  |
| Frequency |  |  |  | <0.001 |
| ≥ 7 times/week | 17939 (67.4) | 16946 (63.7) | 16523 (62.1) |  |
| < 7 times/week | 8672 (32.6) | 9665 (36.3) | 10088 (37.9) |  |
| Duration |  |  |  | <0.001 |
| ≥ 60mins/time | 14177 (53.3) | 12242 (46.0) | 10107 (38.0) |  |
| < 60mins/time | 12434 (46.7) | 14369 (54.0) | 16504 (62.0) |  |

**Table S3. Cross-correlation matrix of the potential confounders.**

|  | Child’s age | Child’s gender | Maternal age at childbirth | Paternal age at childbirth | Monthly household income | Maternal education | Paternal education | Parental history of myopia |
| --- | --- | --- | --- | --- | --- | --- | --- | --- |
| Child’s age | 1.000 |  |  |  |  |  |  |  |
| Child’s gender | -0.016^**^ | 1.00 |  |  |  |  |  |  |
| Maternal age at childbirth | 0.002 | -0.074^***^ | 1.000 |  |  |  |  |  |
| Paternal age at childbirth | -0.005 | -0.048^***^ | **0.713^***^** | 1.000 |  |  |  |  |
| Monthly household income | 0.011 | -0.132^***^ | 0.180^***^ | 0.136^***^ | 1.000 |  |  |  |
| Maternal education | 0.029^***^ | -0.134^***^ | 0.229^***^ | 0.111^***^ | **0.534^***^** | 1.000 |  |  |
| Paternal education | 0.026^***^ | -0.118^***^ | 0.219^***^ | 0.162^***^ | **0.519^***^** | **0.706^***^** | 1.000 |  |
| Parental history of myopia | 0.013^*^ | -0.038^***^ | 0.058^***^ | 0.032^***^ | 0.191^***^ | 0.282^***^ | 0.289^***^ | 1.000 |

*:p<0.05; **: p<0.01; ***: p<0.001

**Table S4. Collinearity evaluation through the VIF among all the variates included in the final model.**

| Covariates | Collinearity Statistics ^a^ |
| --- | --- |
|  | Variance Inflation Factor |
| Child’s gender | 1.000 |
| Child’s age | 1.022 |
| Maternal age at childbirth | 1.024 |
| Monthly household income | 1.073 |
| Parental history of myopia | 1.038 |

a: The closer the Variance Inflation Factor get 1, the lower possibility the collinearity among covariates exists. Usually values of Variance Inflation Factor under 5 show no collinearity among covariates.

**Table S5. Relationship of total screen use from 1 to 3 years of age with preschool myopia**

| Characteristic | All (n=26611) | | Children with non-myopic parents (n=15831) | Children with myopic parents (n=10780) |
| --- | --- | --- | --- | --- |
|  | OR (95%CI) | AOR^a^ (95%CI) | AOR^b^ (95%CI) | AOR^b^ (95%CI) |
| Screen use |  |  |  |  |
| Total screen use |  |  |  |  |
| No | Ref. | Ref. | Ref. | Ref. |
| Yes | 2.41 (1.90, 3.10)*** | 2.50 (1.96, 3.22)*** | 2.85 (1.98, 4.25)*** | 2.27 (1.66, 3.17)*** |

OR: odds ratio; AOR: Adjusted odds ratio; CI: confidence interval; *: p<0.05; **: p<0.01; ***: p<0.001.

^a^ Adjusted for children’s age, gender, maternal age at childbirth, monthly household income, parental history of myopia (myopic, non-myopic), and outdoor activity;

^b^ Adjusted for children’s age, gender, maternal age at childbirth, and monthly household income, and outdoor activity.

**Table S6. Interaction between total screen use and outdoor activity from 1 to 3 years of age on preschool myopia**

| Pattern | | All (n=26611) | |  | Children with non-myopic parents (n=15831) | Children with myopic parents (n=10780) |
| --- | --- | --- | --- | --- | --- | --- |
|  |  | OR (95%CI) | AOR^a^ (95%CI) |  | AOR^b^ (95%CI) | AOR^b^ (95%CI) |
| Total screen use | Frequency of outdoor activity |  |  |  |  |  |
| No | ≥7 times/week | Ref. | Ref. |  | Ref. | Ref. |
| No | <7 times/week | 1.04 (0.64, 1.75) | 1.12 (0.69, 1.89) |  | 1.36 (0.60, 3.67) | 1.04 (0.56, 1.99) |
| Yes | ≥7 times/week | 1.56 (0.99, 2.56) | 1.67 (1.07, 2.75)* |  | 2.26 (0.99, 6.09) | 1.47 (0.87, 2.65) |
| Yes | <7 times/week | 2.83 (1.88, 4.48)*** | 3.17 (2.11, 5.05)*** |  | 4.01 (1.93, 10.25)** | 2.85 (1.76, 4.96)*** |
| Interaction | Screen use (yes)*frequency (<7times/week) | 1.74 (1.00, 2.98)* | 1.69 (0.96, 2.90) |  | 1.30 (0.45, 3.30) | 1.86 (0.92, 3.64) |
| Total screen use | Duration of outdoor activity |  |  |  |  |  |
| No | ≥ 60 mins/time | Ref. | Ref. |  | Ref. | Ref. |
| No | <60 mins/time | 1.23 (0.77, 2.01) | 1.20 (0.75, 1.96) |  | 1.64 (0.77, 3.91) | 1.01 (0.55, 1.88) |
| Yes | ≥ 60 mins/time | 2.33 (1.57, 3.60)*** | 2.47 (1.66, 3.82)*** |  | 3.04 (1.53, 6.94)*** | 2.16 (1.34, 3.69) ** |
| Yes | <60 mins/time | 3.01 (2.06, 4.61)*** | 3.09 (2.11, 4.74)*** |  | 4.59 (2.39, 10.25)*** | 2.46 (1.56, 4.16)*** |
| Interaction | Screen use (yes)*duration (<60mins/time) | 1.06 (0.63, 1.75) | 1.04 (0.62, 1.73) |  | 0.92 (0.37, 2.11) | 1.13 (0.58, 2.17) |
| Total screen use | Overall outdoor factors |  |  |  |  |  |
| No | ≥7 times/week + ≥ 60 mins/time | Ref. | Ref. |  | Ref. | Ref. |
| No | ≥7 times/week + <60 mins/time | 0.81 (0.33, 1.89) | 0.75 (0.31, 1.74) |  | 2.35 (0.46, 17.01) | 0.47 (0.15, 1.29) |
| No | <7 times/week + ≥ 60 mins/time | 0.70 (0.32, 1.53) | 0.73 (0.33, 1.60) |  | 1.73 (0.40, 11.87) | 0.54 (0.20, 1.39) |
| No | <7 times/week + <60 mins/time | 1.07 (0.59, 2.09) | 1.11 (0.61, 2.18) |  | 2.42 (0.70, 15.22) | 0.90 (0.43, 1.97) |
| Yes | ≥7 times/week + ≥ 60 mins/time | 1.34 (0.74, 2.59) | 1.42 (0.78, 2.76) |  | 3.64 (1.01, 23.23) | 1.03 (0.52, 2.17) |
| Yes | ≥7 times/week + <60 mins/time | 1.52 (0.85, 2.93) | 1.52 (0.84, 2.93) |  | 3.70 (1.02, 23.77) | 1.13 (0.59, 2.36) |
| Yes | <7 times/week + ≥ 60 mins/time | 2.23 (1.31, 4.16)** | 2.46 (1.44, 4.59)** |  | 4.83 (1.48, 29.75)* | 2.01 (1.11, 4.03)* |
| Yes | <7 times/week + <60 mins/time | 2.77 (1.66, 5.11)*** | 2.97 (1.76, 5.48)*** |  | 7.41 (2.34, 45.07)** | 2.15 (1.21, 4.24)* |
| Interaction | Screen use (yes)*outdoor activity (<60mins/time+≥7times/week) | 1.40 (0.55, 3.71) | 1.45 (0.56, 3.85) |  | 0.44 (0.05, 2.71) | 2.39 (0.77, 8.38) |
|  | Screen use (yes)*outdoor activity (≥60mins/time+<7times/week) | 2.38 (1.02, 5.59)* | 2.37 (1.01, 5.59)* |  | 0.79 (0.11, 3.92) | 3.57 (1.27, 10.60)* |
|  | Screen use (yes)*outdoor activity (<60mins/time+<7times/week) | 1.94 (0.93, 3.85) | 1.89 (0.90, 3.77) |  | 0.85 (0.13, 3.38) | 2.32 (0.98, 5.34) |

OR: odds ratio; AOR: Adjusted odds ratio; CI: confidence interval; *: p<0.05; **: p<0.01; ***: p<0.001.

^a^ Adjusted for children’s age, gender, maternal age at childbirth, monthly household income, and parental history of myopia.

^b^ Adjusted for children’s age, gender, maternal age at childbirth, and monthly household income.
